# Eskasoni First Nation's transformation of youth mental healthcare: Partnership between a Mi'kmaq community and the ACCESS Open Minds research project in implementing innovative practice and service evaluation

**DOI:** 10.1111/eip.12817

**Published:** 2019-06-27

**Authors:** Daphne Hutt‐MacLeod, Heather Rudderham, Arnold Sylliboy, Mallery Sylliboy‐Denny, Linda Liebenberg, Jeannine F. Denny, Matthew R. Gould, Norma Gould, Margot Nossal, Srividya N. Iyer, Ashok Malla, Patricia Boksa

**Affiliations:** ^1^ Eskasoni Mental Health Services Eskasoni First Nation Nova Scotia Canada; ^2^ ACCESS Open Minds Eskasoni Eskasoni First Nation Nova Scotia Canada; ^3^ ACCESS Open Minds (Pan‐Canadian Youth Mental Health Services Research Network) Douglas Mental Health University Institute Montréal Québec Canada; ^4^ Faculty of Graduate Studies Dalhousie University Halifax Nova Scotia Canada; ^5^ Department of Psychiatry McGill University Montreal Quebec Canada; ^6^ Prevention and Early Intervention Program for Psychosis (PEPP) Douglas Mental Health University Institute Montreal Quebec Canada

**Keywords:** community participation, health promotion, Indigenous community mental health services, suicide, youth mental health, Canada

## Abstract

**Aim:**

ACCESS Open Minds (ACCESS OM) is a pan‐Canadian project aimed at improving youth mental healthcare. This paper describes implementation of the ACCESS OM objectives for youth mental health service transformation within a pre‐existing Fish Net Model of transformative youth mental healthcare service in the First Nation community of Eskasoni, on Canada's east coast.

**Methods:**

We describe an adaptation of the ACCESS OM service transformation objectives through the complementary blending of Indigenous and Western methodologies. This concept of “Two‐Eyed Seeing” is illustrated as central to engaging youth in the community and attending to their mental health needs and wellness.

**Results:**

The ACCESS OM Eskasoni First Nation Youth Space acts as a central location for the site team and its activities, which expand into the rest of the community to facilitate early identification of youth in need. Rapid access to care is promoted via barrier‐free availability through a central intake crisis and referral centre, and ease of contact through social media and other modalities. Youth are given the choice between standard Western mental health services, or Indigenous methods of improving well‐being, or a combination of the two.

**Conclusions:**

The ACCESS OM framework has shown early results of being a positive addition to the Eskasoni community. Local leadership and community buy‐in are identified as key factors to success. Further exploration, research, and evaluation of this transformation is ongoing. Successful implementation of this model in Eskasoni could act as a model for youth mental health programmes in other First Nations across Canada.

## 1 INTRODUCTION

Eskasoni First Nation is a rural Mi'kmaq community located on Cape Breton Island, Nova Scotia, on Canada's east coast, with a total registered population of 4556 (Indigenous and Northern Affairs Canada, 2018). More than 50% of the population is under the age of 25. Thus, provision of health and wellness care to the community's youth is integral to the future of the community. This paper describes the transformation of youth mental health service delivery in Eskasoni in alignment with ACCESS Open Minds (OM), a pan‐Canadian project in which youth mental health services are being transformed to achieve five main objectives: early case identification, rapid access to initial assessment, availability of appropriate services, engagement of youth and families, and elimination of transitions in service based on age (Malla et al., 2018).

In implementing a pan‐Canadian project in our First Nations community, it is of critical importance that the service transformation responds appropriately to the needs of the youth in the community, respecting Mi'kmaq traditions, values, learnings, language, and historical contexts, while integrating the elements of Western knowledge that can support young people in their pathway towards wellness. The “Two‐Eyed Seeing” approach, created by honoured Mi'kmaq Elders Albert and Murdena Marshall, has been a guiding principle for the Eskasoni team and has been integrated into all aspects of service delivery with community youth. This approach is based on the idea that one can learn to take the perspective of seeing from one eye the strengths of Indigenous ways of knowing, and from the other eye, the strengths of Western ways of knowing, and to use both of these perspectives for the benefit of those being served (Bartlett, Marshall, & Marshall, 2007).

At the outset of the ACCESS OM project in 2014, existing initiatives that had been put in place to address youth mental health needs in Eskasoni First Nation lent themselves well to integration with the five ACCESS OM objectives (Malla et al., 2018). ACCESS OM has served to emphasize and accelerate transformational components of youth mental health services in the community, and importantly, allows for systematic evaluation of the effectiveness of these services.

## 2 IMPETUS FOR TRANSFORMATION

During the fall of 2008 and the winter of 2009, the community of Eskasoni First Nation suffered the loss of a number of youth by suicide and accidental drug overdose. The resulting negative news headlines caused emotional, mental and economic suffering to the population. This experience and challenges within the community, such as an increase in prescription and intravenous drug misuse and high rates of poverty (Frank & Saulnier, 2017), mortality and unemployment, exacerbated other systemic and underlying issues such as the intergenerational traumatic effects of the residential school system and other catastrophic impacts of decades of colonial policy promoting cultural discontinuity (Bombay et al., 2019; Kirmayer, Brass, & Tait, 2000; Kirmayer, Gone, & Moses, 2014; Truth and Reconciliation Commission of Canada, 2015; Wilk, Maltby, & Cooke, 2017). A regional health survey at the time reported that between the current issues and persisting community loss, “nearly every young person in the [Eskasoni] community has been impacted negatively,” with 16.5% of youth surveyed expressing that they had contemplated suicide (First Nations Regional Health Survey, 2010).

## 3 PRE‐ACCESS OPEN MINDS TRANSFORMATION

Beginning in 2010, following the 2008 to 2009 suicides and untimely youth deaths, Eskasoni Mental Health Services responded with a process of major transformation of mental health and addictions service delivery in the community. One of the first steps was to amalgamate and co‐locate formerly siloed community mental health services, crisis services and case management into a united and integrated team under one director. This newly reformulated team was given a mandate to provide coordinated, streamlined, barrier‐free and user‐friendly mental health and addiction services. A community‐wide mental healthcare service model—the Fish Net Model—was developed and adopted, which emphasized community development and ownership of programs, and aimed at building upon community‐identified priorities. This model of care is uniquely tailored to the needs, objectives and conceptualizations of health of the community. The approach involves casting a wide net across the community in a variety of ways and for an assortment of interventions. These interventions include standard Western mental healthcare services (e.g., psychology and clinical therapy services, social work, case management, therapy groups); cultural support (e.g., residential school survivor and descendant services, connecting youth with Elder support, providing youth and families opportunities to engage in Mi'kmaq traditions and practices); programming and activities for youth, families and community members of all ages; peer support; and crisis services (see Liebenberg & Hutt‐MacLeod, 2017).

A number of components were woven into the existing Fish Net Community Mental Health model of service delivery in Eskasoni that align well with ACCESS OM objectives, including youth programming to promote early case identification, implementation of barrier‐free and user‐friendly services, and the involvement of youth, families and community members at all levels of care. The Fish Net model also attempted, with no additional funding or support, to fulfil and implement the components of the First Nations Mental Wellness Continuum Framework (2015), jointly developed by the First Nations and Inuit Health Branch of Health Canada, the Assembly of First Nations, and Indigenous mental health leaders from various First Nations. This framework highlights the importance of focusing on all areas of health, including emotional, spiritual, physical and mental wellbeing, and correspondingly hope, belonging, meaning and purpose.

## 4 COMMUNITY MAPPING AND RESEARCH AS A TOOL FOR CREATING TRANSFORMATION

The ACCESS OM project began in Eskasoni in 2014 with numerous community mapping exercises. These exercises were facilitated by Eskasoni's relatively small geographical size allowing for convenient communication among its community services. Community mapping included creating a physical map of services and potential gaps, outlining and dissemination of community events, partnering with Kids Help Phone Interactive map, creating an online portal to accessing services, showcasing initiatives through artistic products and performances, conducting numerous focus groups, creation of videos and staff evolutionary meetings. Community mapping continues on an ongoing basis to ensure that services remains relevant, timely and responsive to the community by continuously identifying and remediating service gaps.

Mi'kmaq culture, tradition and language is deeply rooted within all aspects of Eskasoni's community life. Moreover, cultural connectedness has been reported to be strongly associated with indicators of mental wellness among First Nations youth (Liebenberg & Reich, 2016; Snowshoe, Crooks, Tremblay, & Hinson, 2017). Previous resilience research within the Eskasoni community (Liebenberg, Sanders, & Munford, 2016; Liebenberg, Ungar, & Vijver, 2012) has provided foundational information regarding core elements of youth resilience. These findings are in alignment with the Spaces and Places participatory action research initiative carried out in Eskasoni First Nation (Liebenberg & Reich, 2016; Liebenberg, Wood, & Wall, 2018), which outlined the following themes as pertinent to Eskasoni youth resilience: relational supports (family, Elders, friends and the broader community); engagement with culture, including language and nature; strong personal attributes like self‐esteem; and holistic education. These themes have guided Eskasoni Mental Health Services in adjusting their services with the aim of fostering resiliency among youth accessing these programmes. The idea of centring services around an individual and their family, and their individual and collective needs, has been viewed as central to providing integrated holistic youth mental health services in Eskasoni. Moreover, in a community where youth make up a larger proportion of the population than in the previous generation, the strength of resiliency—and its connection to ancestral knowledge and ways of being and living—are of utmost importance.

## 5 EARLY IDENTIFICATION

The Eskasoni mental healthcare service model contains a wide variety of approaches expressly designed to meet the first ACCESS OM objective, namely to increase the number of youth with mental health problems seeking services through early identification. In Eskasoni, the mental health staff members provide counselling support, facilitate mental health and well‐being groups in the community and organize and facilitate sport, recreation, community and cultural activities. They meet youth and other clients (family members, carers) in the location of their choice—in the family home, in the community, on the land—and are outgoing, approachable and visible individuals within the community and to other community services (e.g., other health and school staff). Their outreach and visibility in the community are essential tools for reducing stigma, breaking down barriers to care and identifying those struggling as early as possible to connect them with appropriate services. The goal is to engage in activities that bring staff and youth together, establish relationships and build trust with the youth, whether they require mental health support or not. Should those youth require more specific mental health services in the future, they will have already forged a trusting bond with the mental health staff.

A wide variety of activities take place at the ACCESS OM Youth Space, renovated through funding from the ACCESS OM project. This acts to reduce stigmatization in a small community where stigma around mental healthcare, though improving, is still present. The community's youth have identified that it is important for them to be viewed in a holistic way, taking into account the balance between emotional, mental, spiritual and physical well‐being outlined in the Medicine Wheel (Bell, 2014), and not to be seen only for their mental health concerns. The Eskasoni service model aims to attract all youth from the community, whether or not they require mental health support. Eskasoni youth see the ACCESS OM Youth Space as a safe and welcoming environment where activities are constantly occurring, and there is no differentiation between mental health programming and regular youth programming. Youth identified activities and programmes that interest them, including movie nights, youth dances, expressive art therapy, song writing circles, piano and guitar lessons, gamer nights, mixed martial arts sessions, exercise classes, traditional Native crafts, regalia making, sweats and language classes. Staff members also share their own Passion Projects (which began as an employee wellness initiative) with youth at the ACCESS OM Youth Space, including gardening, fishing, Wild Child nature programme and baking, to name but a few. Engaging youth in such programmes increases resilience among community youth and the chances of identifying youth in need earlier. Family members and carers often bring their youth to the ACCESS OM space to participate in activities, and then subsequently learn about other available services, or speak with mental health service providers about strategies to best support their young family members. Staff members (Trained Trainers) regularly provide training workshops to community members, especially youth, such as the Mental Health Commission of Canada's Mental Health First Aid for First Nations, Applied Suicide Intervention Skills Training (ASIST) and the Red Cross Healthy Youth Relationships to increase the community's awareness of mental health issues.

## 6 RAPID ACCESS

The ability to access services when needed, and rapidly, is the second objective of the ACCESS OM framework. The underlying principle is that youth in need, or those acting on their behalf, should be able to access someone for a first assessment within 72 hours of first contact, and such access should take place in an engaging environment that does not stigmatize the help‐seeker. In Eskasoni, the methods through which rapid access is promoted include: availability of the ACCESS Clinician, other clinicians and youth peer support workers, use of the youth space and crisis centre, and ease of contact through social media and other modalities. To complement the benefits of the Youth Space, key staff members were added to the team in order to meet the objective of rapid access. As a result, a clinical psychologist, a behaviour interventionist, a youth worker, an intake worker and a research assistant were hired as a crucial part of the ACCESS OM transformation at Eskasoni. The ACCESS OM Research Assistant/Intake worker facilitates rapid access in two different ways: first, the intake and assessment process is accelerated as the worker obtains demographics and administers scales (e.g., Kessler Psychological Distress Scale, K10) to obtain initial clinical information from the youth; second, evaluation data is made available on a regular basis, providing timely information on emerging needs of community youth. For example, when data indicated trends of increased self‐harm among youth seeking services, Eskasoni Mental Health Services responded with increased visibility of services through social media, public service announcements on local cable television, and school presentations, and held a staff consultation with a leading Nova Scotia self‐harm care provider at the regional children's hospital.

Additionally, access to Eskasoni's services were largely barrier‐free prior to ACCESS OM implementation; numerous modalities exist to contact support workers and service providers within the community, such as a toll‐free telephone number and various social media platforms (including Messenger, Twitter, Facebook). Youth in Eskasoni also have rapid access to support through the Crisis/Distress/Central Intake and Referral Centre, which is open 24 hours a day, 7 days a week, year‐round. Crisis staff are trained to identify individuals who may benefit from additional services and offer connection to those services. Although the Crisis Centre is funded through Eskasoni Band Own Source Fishing Revenue, and is not an ACCESS OM initiative, these staff work directly with ACCESS OM staff to facilitate connecting youth to support services, central intake and crisis intervention.

## 7 APPROPRIATE CARE

Appropriate care is provided in accordance with the “Two‐Eyed Seeing” approach (see above). In consultation with the clinician, youth are given the choice to have a standard Western mental health service, or an Indigenous method of improving well‐being, or any combination of the two that they prefer. The Eskasoni team's Ladder of Care, which includes peer supporters with lived experience, paraprofessionals, baccalaureate, graduate and post‐graduates, will often connect youth with multiple services, programs and providers. This approach allows youth seeking support to have access to a wide range of helping professionals (psychologists, social workers, family physicians), paraprofessionals (with certificates and diplomas in mental health and addictions), youth peer support, well‐being activities and groups and supportive community members. Family physicians work collaboratively with the mental health practitioners in instances where youth require psychiatric medication. Indigenous traditions of improving mental wellness that may be offered include working with Elders in the traditional medicine garden, participating in land‐based nature programmes and summer culture camps, taking part in traditional pipe ceremonies, sweat lodge ceremonies, naming ceremonies, Grandmother Moon ceremonies, blanket ceremonies and Letting Go ceremonies, and practicing traditional crafts such as drum making, beadwork and basket‐making. This team approach, wherein youth have access to a range of supports, more effectively addresses the appropriate care of youth in need; not every young person experiencing distress necessarily requires extensive psychological services. Depending on circumstances, the care that a young person needs comes in different forms, whether it is reducing isolation, connecting to the land and one's culture, or a discussion with a counsellor in times of grief. For example, a young person may meet with a psychologist for in‐office therapeutic services, as well as a youth peer supporter who is available between appointments for added support, or to help connect them with community programmes or services. Dedicated on‐going communication between the support team and the youth is a key component to providing this approach to care.

A challenge is posed in providing appropriate care when youth require referrals for more specialized services, such as those of a psychiatrist, not available within the community. This remains difficult due to limited resources in the provincial healthcare system. The local health authority is experiencing a significant shortage of psychiatrists that has led to long wait times and difficulty connecting youth to these specialized services. Any local youth requiring specialized mental health service is required to travel four and a half hours to the hospital in Halifax to obtain services. This can impose a major stressor on an already vulnerable youth who may have to travel and remain in Halifax without an accompanying family member due to financial or other constraints. Use of tele‐health services for accessing psychiatry and other specialized mental health services is an option that is being explored.

## 8 CONTINUITY OF CARE

Another important objective within the ACCESS OM framework is the elimination of disruptions in service based on age when youth transition from youth to adult services. Instead, transitions are based on needs and seek to encourage prompt, seamless and continuous access to care. In Eskasoni, this objective of elimination of age‐based disruptions in service was already met prior to the ACCESS OM project. The aforementioned amalgamation drew multiple services under one team umbrella and mental health services are offered from “womb to tomb,” meaning that the whole community is served across the lifespan. A client who has built a relationship with a clinician or service provider over time does not need to worry about having to “start over” with a new clinician or being discharged from services because they have “aged out.” Transitions to a new service or another provider are based on the client's need and desire rather than age restrictions.

## 9 YOUTH AND FAMILY ENGAGEMENT

Meaningful engagement of youth and their families and carers has been a key principle and objective of ACCESS OM nationally, and is readily embodied in Eskasoni, assuring that the services created are inviting, effective and cater to the needs and preferences of youth in the community. Such engagement is likely to increase participation in services, decrease drop‐out rates and maximize benefits for service users. Several methods have been used to engage youth locally. A local youth council was created to design the ACCESS OM Youth Space, and continues to contribute to the creation of programming, activities and services. ACCESS OM team members regularly visit the local schools, seeking feedback on current services and programs, and gathering suggestions for future activities. The ACCESS OM Youth Space also provides programmes meant to engage entire families. For example, the ACCESS OM Youth Space hosts parenting programmes and “special needs” support groups facilitated by ACCESS OM and Eskasoni Mental Health Services team members.

One transformation that occurred in service provision in response to youth feedback was broadening means of communication and removing barriers to accessing services. Youth expressed that they would like to be able to self‐refer online, and reach out through social media to begin the process of engaging in services. As a result, the site team uses social media such as Facebook to advertise events at the Youth Space, online messaging to communicate with youth, Instagram and Twitter to share photos and news, and maintains a website to reach a wider audience. Eskasoni is also currently working to launch an online self‐referral platform.

## 10 RESEARCH AND EVALUATION

Since July 2016, the Eskasoni First Nation team has been carrying out the ACCESS OM Evaluation Protocol, recruiting youth receiving services and engaging them in providing feedback on outcomes and satisfaction with services, both on the clinical/individual level, as well as at a site‐level. To promote integration of the ACCESS OM evaluation into the entire mental health service, the entire site team participated in the initial knowledge sharing and training sessions facilitated by the ACCESS OM central office team. As well, many of the site team members perform data collection and entry using Dacima software, the online electronic database system that the network is using to manage data. Evaluation data provides information on emerging needs and will allow our site to evaluate the quality of mental health care offered, identifying interventions effective at improving youth mental health. Participation in the ACCESS OM evaluation may also provide a powerful tool for lobbying for sustainable funding and policy change related to youth mental health in First Nations communities.

## 11 DISCUSSION

One of the advantages of being a part of the pan‐Canadian ACCESS OM initiative is that while all sites aim to achieve the same objectives, each site is also encouraged to customize the implementation to unique site populations. The ACCESS OM Eskasoni First Nation team takes a “human first” mentality; foundational to everything is to meet people where they are (physically, geographically, socially, spiritually and emotionally), engage them in what interests them, and teach and learn about wellness from a holistic perspective. Culture is embraced as the underpinning of mental wellness programmes, addressing the need for cultural competency and humility embedded within service delivery (Kirmayer et al., 2000; Kirmayer, Tait, & Simpson, 2009). Through an integrated Two‐Eyed Seeing approach, both Indigenous‐ and Western‐influenced methods of wellness and treatment are implemented and honoured, providing youth access to culturally appropriate and engaging mental health services. An emphasis on promotion, prevention, education and community involvement in these culturally competent and client‐centred services aims to strengthen youth resilience and to promote early identification and engagement of young people at risk of progressing to development of a mental disorder. Within a First Nations framework, helping those with even complex mental health needs is inextricably linked with the holistic promotion of mental wellness. As stated in the First Nations Mental Wellness Continuum Framework (2015): “Mental wellness is supported by culture, language, Elders, families and creation and is necessary for healthy individual, community and family life.”

As with any time‐limited funded research project, an ongoing challenge to the ACCESS OM team in Eskasoni is finding long‐term funding for the transformations taking place. Sustainability is a constant pressure. Many mental health initiatives encountered in First Nations communities in Canada are obtained via demonstration sites, pilot projects or proposal driven initiatives. Limited by staff who also provide front‐line services, the Eskasoni team is in a constant state of writing and submitting proposals to granting agencies and charitable organizations, to supplement staff salaries and funding of programming. This is a systemic problem with the way in which Indigenous communities are funded for mental health services in Canada (mainly from a combination of federal, provincial and local Band funding). Additional challenges are caused by the fact that with increased visibility in the community comes an increase in youth seeking mental health services, which can challenge the capacity of the service. Lastly, an outstanding challenge is around family/carer engagement. Although youth present more readily for services, and it would seem that stigma has been reduced in general, many youth are still hesitant to involve family members in their care journey. The site team aims to address ways to support families and carers while still honouring the wishes of youth and maintaining confidentiality. The ACCESS OM team in Eskasoni regularly seeks feedback from youth service users, youth council members, their support systems and the community on ways to improve services.

While issues of sustainability, program funding, scale‐up and capacity of service providers to meet service demands remain a constant challenge, a major strength has been the ability of the Eskasoni youth mental health team to attract staff who are enthusiastic, competent, dedicated, passionate, and who really want to work in Eskasoni. The Eskasoni First Nation team aims to support other Indigenous communities undertaking similar transformations, and hopes to act as a model for future communities who seek to improve mental healthcare services for their youth.
